# An Overview of Antitumour Activity of Polysaccharides

**DOI:** 10.3390/molecules27228083

**Published:** 2022-11-21

**Authors:** Hongzhen Jin, Maohua Li, Feng Tian, Fan Yu, Wei Zhao

**Affiliations:** 1 College of Pharmacy, Nankai University, 38 Tongyan Road, Jinnan District, Tianjin 300350, China; 2 College of Life Sciences, Nankai University, Weijin Road, Nankai District, Tianjin 300350, China; 3 State Key Laboratory of Medicinal Chemical Biology, Nankai University, 38 Tongyan Road, Jinnan District, Tianjin 300350, China

**Keywords:** anticancer, polysaccharides, drug delivery systems, nanomedicines

## Abstract

Cancer incidence and mortality are rapidly increasing worldwide; therefore, effective therapies are required in the current scenario of increasing cancer cases. Polysaccharides are a family of natural polymers that hold unique physicochemical and biological properties, and they have become the focus of current antitumour drug research owing to their significant antitumour effects. In addition to the direct antitumour activity of some natural polysaccharides, their structures offer versatility in synthesizing multifunctional nanocomposites, which could be chemically modified to achieve high stability and bioavailability for delivering therapeutics into tumor tissues. This review aims to highlight recent advances in natural polysaccharides and polysaccharide-based nanomedicines for cancer therapy.

## 1. Introduction

In the coming years, cancer is expected to become the main cause of death and the most important obstacle to extending life expectancy in the world. Lung cancer is the most common cancer and the leading cause of cancer death (18.4% of total cancer deaths), closely followed by colorectal cancer (9.2%), stomach cancer (8.2%), and liver cancer (8.2%) [1]. There are three common cancer therapeutics, including surgery, radiation therapy, and chemotherapy, as well as other emerging therapies, such as molecular targeted therapy. However, the serious side effects and drug resistance of chemotherapy and other treatments are becoming major obstacles in current cancer research. Hence, it is very important to develop a new type of anticancer agent with ideal antitumour activity and extremely low toxicity.

Polysaccharides are carbohydrates that participate in almost all aspects of organisms and play various important biological functions [2]. Polysaccharides consist of 10 or more monosaccharides linked together by glycosidic bonds, which can be linear or contain branched chains. Importantly, monosaccharide composition, molecular weight (MW), and polysaccharide attachment affect its structure, and its structure further affects its properties and functional mechanisms [3]. According to their source, polysaccharides can be classified into natural polysaccharides and semisynthetic polysaccharides. Natural polysaccharides are distributed in many organisms. Then, the natural polysaccharide is further chemically or enzymatically modified to obtain semisynthetic polysaccharides. So far, researchers have found that polysaccharides have a wide range of biological effects, including anticancer, antibiotic, antioxidant, anticoagulant, and immuno-stimulation activities.

The antitumor effect of polysaccharides was first discovered by Nauts et al. in 1946, which can effectively relieve the symptoms of cancer patients [4]. Ample evidence indicated that polysaccharides can inhibit tumors through direct anticancer activity, such as inducing apoptosis of tumor cells and inhibiting migration (Table 1). In addition, the structure of polysaccharides provides versatility for the synthesis of multi-functional nanocomposites, which can achieve high stability and bioavailability through chemical modification, thus delivering therapeutic drugs to tumor tissues [5]. This review used keywords (anticancer/polysaccharides/drug delivery systems/nanomedicines) to search in PubMed and Web of Science databases, and selected qualified high-level papers for systematic sorting and summary. In this paper, we aim to systematically summarize the research findings in the past decade, and the different structures of anticancer polysaccharides from different sources and polysaccharide-based nanomedicines for cancer treatment are reviewed, which provides theoretical support for the design and development of polysaccharide preparations.

## 2. Polysaccharides from Plants

### 2.1. *Panax ginseng C. A. Meyer* Polysaccharides

*Panax ginseng C. A. Meyer* (*P. ginseng*) is a precious medicine that has been used for thousands of years, also known as ginseng [6]. Ginseng is composed of multiple active components, including ginsenosides and polysaccharides. Studies have proven that polysaccharides are one of the most important components in *P. ginseng* and participate in immunomodulation, antitumour, and antidiabetic activities [7].

*P. ginseng* polysaccharide contains starch-like glucans and pectin [8]. Pectin is a plant-derived neutral polysaccharide with abundant resources for its amounts and categories. Many types of pectin polysaccharides are associated with anticancer activity. Pectin, with Panax ginseng C. A. Meyer Polysaccharidesvery complex structure, typically contains galacturonic acid (GalA), galactose (Gal), arabinose (Ara), and rhamnose (Rha) residues [9]. Pectin could be divided into five types: homogalacturonan (HG), type I rhamnogalacturonans (RG-I), type II rhamnogalacturonans (RG-II), xylagalgalacturonan (XGA), and Apio galgalacturonan (AGA), based on the different structural characteristics [10]. HG is characterized by α-(1→4)-D-GalA repeat units as the backbone [11], whereas RG-I is composed of Ara, galactans, and L-fucose (L-fuc) in the sidechains [12]. RG-II and XGA are both derivatives of HG [10]. The components of P. ginseng pectin include HG and RG-I, as well as GalA, Gal, Ara, and Rha [13].

To date, many kinds of pectin have been isolated and identified from ginseng, and some of them have been identified as having antitumour activity, as described in Table 2.

Ginseng polysaccharide could also significantly inhibit the growth of Lewis lung carcinoma tumor [19]. In addition, one selenium-modified polysaccharide, sGP, has been reported. The experimental results indicate that sGP enhances apoptosis in HL-60 cells, demonstrating that chemical modification methods to obtain high contents of selenium polysaccharides could be developed as a novel antitumour therapy [20].

### 2.2. *Angelica Sinensis* (Oliv.) Diels Polysaccharides

The root of *A. sinensis*, known as Danggui, is a celebrated Chinese medicinal herb [21]. *A. sinensis* possesses a wide range of pharmacological activities, including hematopoiesis, immunomodulation, antioxidant, and anticancer activities [22,23,24,25]. Polysaccharides are the most important active constituents in Danggui, and numerous *A. sinensis* polysaccharides (ASPs) have been identified. The majority of ASPs contain GalA, Gal, Ara, Rha, mannose (Man), and glucose (Glc) with various molar ratios. Wei et al. also proved that APSs could induce apoptosis in cancer cells via regulation of the JAK/STAT of the transcription pathway [26]. Key kinases in the JAK/STAT and PI3K/AKT pathways were also downregulated by ASPs’ stimulation in another study [27]. ASPs have also been utilized in drug delivery systems. Wang et al. prepared doxorubicin (DOX)-loaded nanoparticles and proved that it can inhibit the growth of HepG2 multicellular spheres [28].

### 2.3. *Portulaca oleracea* L. Polysaccharides

*P. oleracea* L., a traditional Chinese herbal medicine, is known as MaChiXian in Chinese and purslane in English. It exhibits a range of biological activities, such as anti-inflammatory, antioxidant, and antiaging [29,30,31,32]. *P. oleracea* L. polysaccharides (POL-P) are major bioactive components of purslane with antitumour activity. Zhou et al. purified a homogeneous POL-P, which contains Gal, Ara, Man, and Glc. Then, they evaluated an animal model transplanted with sacroma 180 and found that it had pronounced antitumour effects [33]. Another POL-P, named POL-P3b, inhibits cancer cell growth, and the mechanism involves triggering DNA damage and inducing apoptosis [34]. Further research also showed that POL-P3b inhibits the proliferation of HeLa cells, and the possible antitumor mechanism is through downregulating the TLR4 downstream signaling pathway and inducing cell apoptosis [35]. In addition, POL-P3b could also decrease the growth of cervical carcinoma, suggesting the antitumour mechanism via stimulating the TLR4/PI3K/AKTNF-κB signaling pathway [36].

In addition to direct antitumour effects, Lee et al. go deeply into the immune-enhancing characteristics of POL-P. The preliminary results showed that POL-P increased the viability of CY-treated splenocytes because of CY-induced immunosuppression [37]. POL-P also enhances the immune efficiency of the breast cancer dendritic cell vaccine [38]. Ding et al. found that POL-P can improve lipopolysaccharide-induced inflammation and barrier dysfunction of the porcine intestinal epithelium monolayer [39].

### 2.4. *Lycium barbarum* L. Polysaccharides

*L. barbarum*, known as wolfberry in China, is a herbal medicine [40]. Polysaccharides are one of the most investigated, as they are considered to be mainly responsible for different biological effects among all *L. barbarum* components [41]. Zhao et al. extracted polysaccharides from Chinese wolfberry fruits and proved that it could induce MCF-7 cell apoptosis. Cao et al. isolated and characterized another polysaccharide, named CF1, with an MW of 1540.10 ± 48.78 kDa. Their results showed that CF1 also exhibited effective cell growth inhibition in vitro [42]. Then, Cao et al. conducted further research and exploration. Eventually, they found that the antitumour mechanism of CF1 was associated with the PI3K/AKT pathway [43].

### 2.5. *Ginkgo biloba* Polysaccharides

*G. biloba*, known as yinxing in China, is a traditional Chinese herb. Polysaccharides are bioactive compounds isolated from *G. biloba*, with a wide variety of physiological functions such as antitumor activity. Kong et al. reported a selenium (Se)-containing polysaccharide purified from the leaves of *G. biloba*, and proved that it induced human bladder cancer T24 cell apoptosis through a mitochondria-dependent pathway [44].

### 2.6. Seeds’ Polysaccharides

Seeds are one of the important sources of plant polysaccharides and accumulated evidence has demonstrated that these polysaccharides show superior anticancer activity, as described in Table 3.

### 2.7. Citrus Polysaccharides

Citrus pectin is a neutral polysaccharide isolated from the pulp and peel of citrus fruits, which consists of HG and RG-I [48]. Modified citrus pectin (MCP) is a nonbranched polysaccharide and is high in Gal extracted from citrus pectin by enzymatic hydrolysis, high temperature, and high pH [49]. The shorter and nonbranched MCP could recognize and bind tightly with galectin-3 [50], whose overexpression was related to a variety of malignant tumors [51]. The combination mechanism of MCP and galectin-3 is that the former can recognize galectin-3 on the surface of cancer cells and then inhibit tumor metastasis [49,50]. However, citrus pectin from a neutral resource is unable to interact with galectin-3 owing to its limited solubility in water.

It has been reported that MCP inhibits myeloma/prostate cancer/bladder tumor [52]/gastrointestinal cancer [53] via interaction with galectin-3. Conti et al. found that MCP is a potential sensitizer targeting galectin-3 for prostate cancer radiotherapy [54]. Fabi et al. demonstrated that MCP fractions with different molecular sizes can have different effects on the development of malignant tumors [55]. In addition, pectic from Aegle marmelos L. could potentially inhibit skin cancer [56]. Additionally, pectin polysaccharides extracted from tomato, papaya, or olive have been reported to possess the activity of inhibiting galactose lectin-3. The pectin polysaccharide fraction from papaya pulp and olive showed inhibitory effects on colon cancer [57] and bladder cancer [58], respectively, through interactions with galectin-3.

### 2.8. Marine Algae Polysaccharides

**Figure 1:**
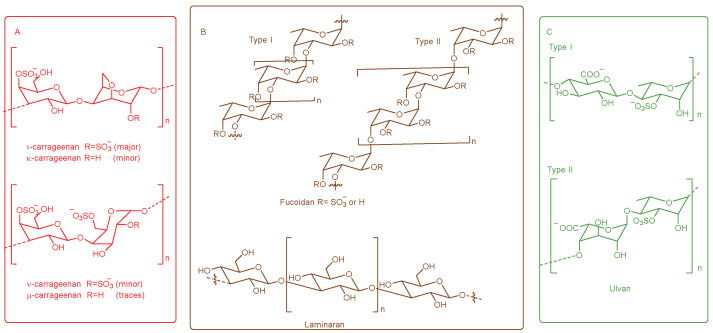
The major MAP in red seaweed (**A**), brown seaweed (**B**), and green seaweed (**C**).

Marine algae are one of the richest resources in the ocean, and contain a variety of active components, such as peptides and polysaccharides [59]. According to the thallus color, marine algae are usually divided into red seaweed, brown seaweed, and green seaweed. Marine algal polysaccharide (MAP) is a unique polysaccharide, which is different from land plant polysaccharides in composition, substitution, and linkage [60]. The major MAP contains carrageenan of red algae, fucoidan and laminarans of brown algae, and ulvan of green algae, comprising monosaccharide subunits such as Gal, Ara, Glc, Man, fucose, xylose, glucuronic acid (GlcA), mannuronic acid (ManA), and iduronic acid (IdoA) [61,62] (Figure 1).

According to a previous study, polysaccharides fractionated from brown seaweed Sargassum (S.) show superior anticancer activity. For example, a study showed that sulfated polysaccharides could inhibit proliferation in A549 cells via induced mitochondria-mediated intrinsic apoptosis and cell cycle arrest [63]. Rajendran et al. obtained polysaccharide fractions (SWP1) from *S.* wightii and found that it showed a dose-dependent manner inhibition of proliferation and migration of cancer cells. Further research reveals that the mechanism of SWP1 inducing apoptosis in cancer cells is via cutting the mitochondrial membrane and damaging the nucleus, as well as increasing caspase 3/9 activity [64]. Fucoidan, a sulfated polysaccharide rich in fucose, has antitumour activities [65]. The experimental results of Kang et al. also prove that fucoidan possesses anti-proliferation of B16 melanoma cell [66]. Alginate oligosaccharide was prepared from alginate sodium using alginate lyase and can reduce tumor size by improving the antioxidant and anti-inflammatory capacities of patients [67]. The red seaweed sulfated polysaccharide from Acanthophora spicifera (Vahl) Borgeson exhibited apoptotic effects in lung cancer cells [68]. In addition, polysaccharides isolated from two microalgae sources showed certain ant-hepatoma activity in vitro mainly through the induction of apoptosis [69,70].

### 2.9. Other Plant Sources of Polysaccharides

#### 2.9.1. Polysaccharides with Anti-Lung Cancer Activity

Ni et al. successfully separated HRWP-A, a natural pectin, from Hippophae rhamnoides berries. HRWP-A effectively inhibits the growth of lung cancer in vivo and promotes NK cell activity and CTL mechanism by enhancing lymphocyte proliferation and macrophage activity [71]. HCA4S1 was separated from Houttuynia cordata, and bioactivity tests suggested that it exerts anticancer action via inducing cell cycle arrest and apoptosis on lung cancer cells [72]. Additionally, Glehnia littoralis polysaccharide effectively inhibits the proliferation and migration of A549 cell lines and induces cell apoptosis [73]. Lee et al. showed that the bioactive polysaccharides from Achyranthes bidentata exhibit potential anti-metastasis effects with the mechanisms of blocking the epithelial-to-mesenchymal transition process [74].

#### 2.9.2. Polysaccharides with Anti-Pancreatic Cancer Activity 

*Lonicera japonica* and *Lycium ruthenicum* pectin have certain inhibitory effects on pancreatic cancer in vitro. LJ-02–1 is an RG-I polysaccharide, and bioactivity tests suggested that it might inhibit BxPC-3 and PANC-1 cell growth [75]. LRP3-S1 could also inhibit the growth of pancreatic cancer cells via downregulating the protein expression of p-FAK and p-p38 MAP kinase [76].

#### 2.9.3. Polysaccharides with Anticancer Activity 

In addition to lung cancer and pancreatic cancer, polysaccharides from other species of plants have also been reported for the use of other malignant tumors, as shown in Table 4.

## 3. Polysaccharides from Animals

### 3.1. Polysaccharides from Mammals

**Figure 2:**
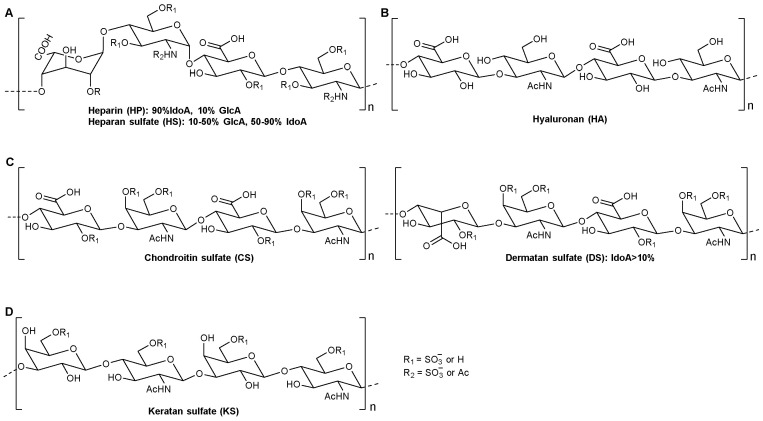
Four classes of mammalian GAGs and their potential sulfation sites. (**A**) (HP)/heparan sulfate (HS), (**B**) Hyaluronan (HA), (**C**) Chondroitin sulfate (CS)/Dermatan sulfate (DS), and (**D**) Keratan sulfate (KS).

Glycosaminoglycans (GAGs) are natural linear polydisperse heteropolysaccharides distributed in both vertebrates and invertebrates, with molecular weights up to several million Dalton [85]. Evidence obtained from glycobiology studies suggests that GAGs can recognize and interact with numerous proteins, and thus possess extensive biological functions [86]. GAGs are one class of glycostructures of the extracellular matrix (ECM). There are four classes of GAGs, each according to the constitution of the repeating disaccharide units, which consist of heparin (HP)/heparan sulfate (HS), hyaluronan (HA), chondroitin sulfate (CS)/dermatan sulfate (DS), and keratan sulfate (KS) (Figure 2) [85,87]. Except for HA, other compounds contain O-sulfonation, N-acetylation, and N-sulfonation modifications, and this polyanionic character allows GAGs to bind to positively charged moieties, including plasma proteins, growth factors, and so on [87]. These molecules are a kind of ubiquitous molecule with extensive biological functions and, of course, they are also widely used as therapeutics, for example, HP is an anticoagulant, while CS is generally used to treat osteoarthritis [88]. In addition, further understanding of GAG’s structure–function relationships has also led to the discovery of novel pharmaceuticals for the possible treatment of serious diseases, such as antitumor agents. In light of GAGs related to tumorigenesis, its application in drug development has been the focus of two main directions: (I) using GAGs as the target of therapeutic strategies and (II) utilizing the specificity and excellent physical and chemical properties of GAGs to deliver targeted cancer drugs [89].

#### 3.1.1. Heparin/Heparan Sulfate

HP has been used as an anticoagulant for more than 80 years, and it is a true biologic and can be purified from bovine lung or porcine mucosa. The anticoagulant activity of HP is mostly owing to the action of a precise pentasaccharide sequence that acts in accordance with antithrombin-III (AT-III), a serine protease inhibitor [90]. As an important member of the linear GAG family, HP and HS are composed of sulfated disaccharide repeating units of either GlcA- or IdoA-linked glucosamine (GlcN) residues (Figure 2A). HP is, on the whole, more highly sulfated than HS. Depending on the sources and molecular weight differences, HP is classified into the following three classes: (I) unfractionated heparin (UFH), extracted from many animal sources, with an MW of approximately 14,000 Da; (II) low molecular weight heparin (LMWH), prepared from UFH, with a MW of approximately 3500~6000 Da; and (III) ultralow molecular weight (ULMWH), generally referring to the chemically synthesized pentasaccharide fondaparinux sodium, with the trade name Arixtra.

HP, including UFH and LMWH, is used in the treatment of cancer-associated venous thromboembolism (VTE), and LMWH is recommended as the nursing standard for the treatment of established VTE [91,92,93]. Preclinical data support that coagulation inhibition greatly limits tumor metastasis in some experimental models, and it has been demonstrated that LMWH can effectively inhibit metastasis of solid malignant tumors [94]. In addition to anticoagulant activity, HP may possess direct anticancer benefits because of its antiangiogenic properties [95]. The antiangiogenesis mechanism is that HP binds to vascular endothelial growth factor (VEGF) and then inhibits the phosphorylation of VEGF receptor (VEGFR) [96]. Furthermore, HP is an inhibitor of heparanase, which is overexpressed in tumors, and heparin can bind with P-selectin to significantly inhibit tumor cell adhesion [97,98]. As natural resourced polysaccharides, HP are often described as nonimmunogenic and nontoxic, driving the desire to employ them in nanoformulations for cancer management. Because of the above factors, HP plays an important role in cancer treatment, as shown in Table 5.

#### 3.1.2. Hyaluronan

**Figure 3:**
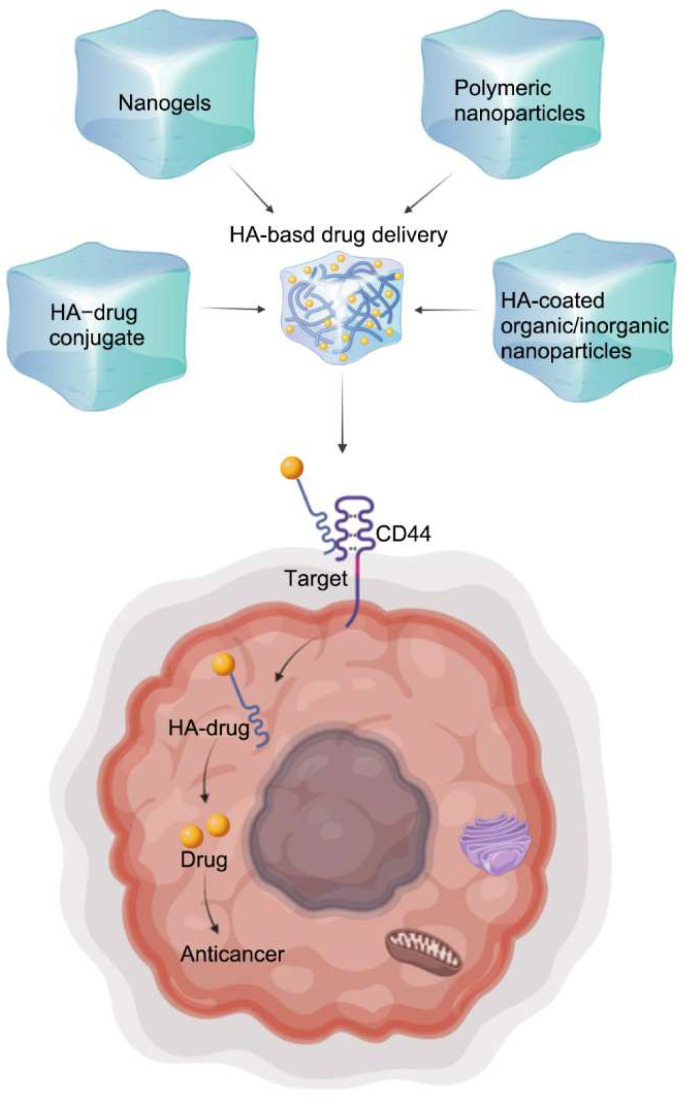
Mechanism of action of HA-based drug delivery targeting CD44.

HA normally exists in the form of long-chain nonsulfated polysaccharides, which are the main component of the ECM in cells [117]. The repeated disaccharide unit of HA is composed of GlcA β (1→3) GlcNAc, and each disaccharide unit passes through a β (1→4) glycosidic bond (Figure 2B). Native HA, extracted from many animal sources, is present as a linear polymer with an average molecular weight of approximately 106~107 Da [118]. Likewise, HA with strong hydrophilicity could form a very viscous gel that helps to maintain tissue integrity [119]. In addition to being a structural part of tissues, HA is the ligand of the cluster of differentiation (CD) protein CD44 receptor [118]. CD44 is a complex transmembrane receptor protein that is overexpressed by many tumor types [117]. Hence, specific ligation with HA-CD44 enables HA-based drug delivery (containing HA–drug conjugates, nanogels, polymeric nanoparticles, and HA-coated organic and inorganic nanoparticles) to target diseased cells that express these receptors (Figure 3). In addition, HA combined with drugs or drug carriers could solve some solubility problems [118].

To date, HA has been widely used in anticancer drug delivery, either associating HA with drugs to form conjugates or producing hydrogels, for the local delivery of various drugs, including antitumoral agents, owing to its biocompatibility, biodegradability, nontoxicity, nonimmunogenicity, and as a ligand of CD44. The application of these nanoparticles in various cancer therapies is shown in Table 6.

#### 3.1.3. Chondroitin Sulfate/Dermatan Sulfate

The repeated disaccharide unit of CS is comprised of GlcA β (1→3) GlcNAc, and each disaccharide unit passes through a β (1→4) glycosidic bond (Figure 2C). CS can be divided into five types according to their different modification types and sulfonation forms, as shown in Table 7 [87,139]. After rare C5 isomerization of CS GlcA into IdoA, a special type CS-B of CS, DS, is produced (Figure 2C). As with other GAGs, CS is a special anionic acid polysaccharide with high biocompatibility and specificity, and is a promising drug carrier for cancer treatment.

Curcumin-loaded CS/chitosan nanoparticles inhibited the apoptosis of lung cancer cells, whereas loading CS/chitosan hydrogel with curcumin exhibited cytotoxicity-inducing effects in HeLa, HT29, and PC3 cancer cells [140,141]. Curcumin-loaded zein and CS self-assembled nanoparticles also exhibited anti neoplastic activity on HepG2, MCF-7, and HeLa cells [142]. In colorectal cancer cells, folate-targeted nanostructured chitosan/CS complex carriers, CS–chitosan nanoparticle carriers encapsulating black rice anthocyanins, and CS-based smart hydrogels could heighten the delivery of antitumor drugs to tumor cells [143,144,145,146].

Similar to HA, CS has a great targeting ability for the cluster CD44, which is overexpressed in particular cancer cells [147]. Therefore, the surface functionalization of CS-endowed nanoparticles has been successfully used for the treatment of colon cancer [148]. Moreover, a codelivery vector including CS loaded with small interfering RNA and paclitaxel has been proven to have a mighty targeting effect towards CD44-overexpressing cancer cells [149]. CS-based multi-walled carbon nanotubes can precisely target CD44 receptors overexpressed on triple-negative breast cancer specific cells [150]. In addition, combined application of CS with doxorubicin or quercetin (chemicalsensitizer) can enhance chemical photodynamic therapy and overcome multidrug resistance [151,152].

#### 3.1.4. Keratan Sulfate

KS is localized in the ECM of different tissues, has a relatively small molecular weight, and ranges from 5 to 30 repeating disaccharide subunits. KS is composed of Galβ(1→4)GlcNAc, and each disaccharide unit passes through a β (1→3) glycosidic bond. It is different from other GAGs because its uronic acid moiety is partially replaced by neutral Gal units (Figure 2D) [87]. As a class of GAGs, the potential of KS in the delivery of anticancer drugs needs to be further developed.

### 3.2. Polysaccharides Derived from Marine Animals

#### 3.2.1. Chondroitin Sulfate from Sturgeon and Cartilage

As mentioned earlier, CS is a natural polymer and is widely distributed in the cartilage and bone of animals. Herein, a sturgeon (*Acipenser*)-derived CS significantly inhibits tumor progression of HCT-116 mice model by inhibiting proliferation and inducing apoptosis [153]. Moreover, a novel CS-E exhibits dose-dependent antimetastatic activity [154].

#### 3.2.2. Sulfated Polysaccharides from Sea Cucumber

Sulfated polysaccharides are one of the main components of sea cucumber, which have a wide range of biological activities. Ermakova et al. isolated sulfated fucans and proved that it exhibits anticancer activity against the cancer cell lines [155].

#### 3.2.3. Polysaccharides from Common Cockles

Research by Pye et al. shows that the sulfated polysaccharide has antiproliferative activity on chronic myeloid leukemia and relapsing acute lymphoblastic leukemia cell lines. They identified that sulfated polysaccharides are a unique marine-derived HP/HS-like polysaccharide [156].

## 4. Polysaccharides from Fungi

### 4.1. Lentinan

**Figure 4:**
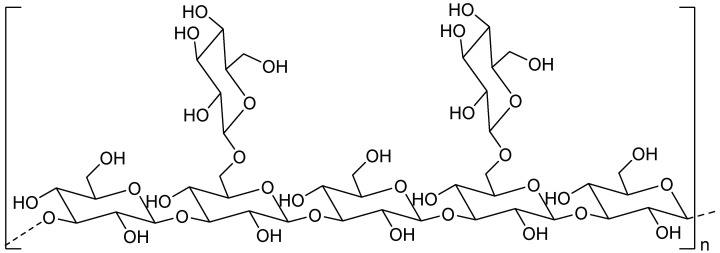
The repeating unit of the LNT structure.

Lentinan (LNT), a neutral polysaccharide extracted from Lentinus edodes, has been widely used in Asia. LNT is a kind of β-(1→3)-D-glucan and its repeating unit is shown in Figure 4. The primary structure of LNT consists of two lateral β-(1→6) glucose branches on five β-(1→3) glucose linkages [157].

**Figure 5:**
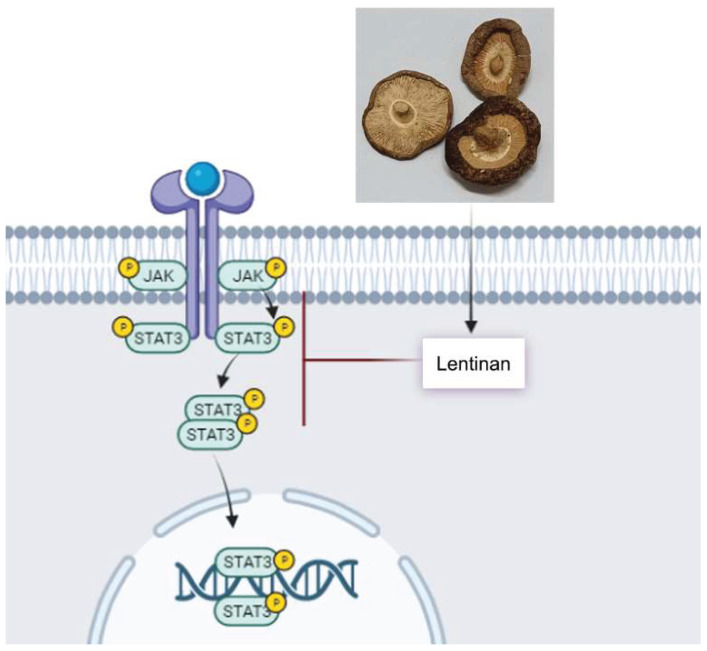
The mechanism action of the Lentinan.

The antitumour activity of LNT and its synergistic effect with various chemicals or other therapies have been extensively studied. Wu et al. reported that LNT can effectively delay the development of lung adenocarcinoma by upregulating miR-216a-5p and inhibiting the JAK2/STAT3 signaling pathway (Figure 5) [158]. LNT as an adjuvant has been prepared into lentinan calcium carbonate (LNT-CaCO_3_) microspheres and has potential use as a vaccine delivery system [159]. Chen et al. used LNT as a modifier to synthesize stable and efficient selenium nanoparticles (SeNPs), which can effectively inhibit the growth of solid tumors [160]. Additionally, LNT-coated selenium nanoparticles (SeNPs@LNT) could restore the dysfunctional immune cells in the malignant pleural perfusion microenvironment [161].

### 4.2. *Ganoderma lucidum* Polysaccharide

**Figure 6:**
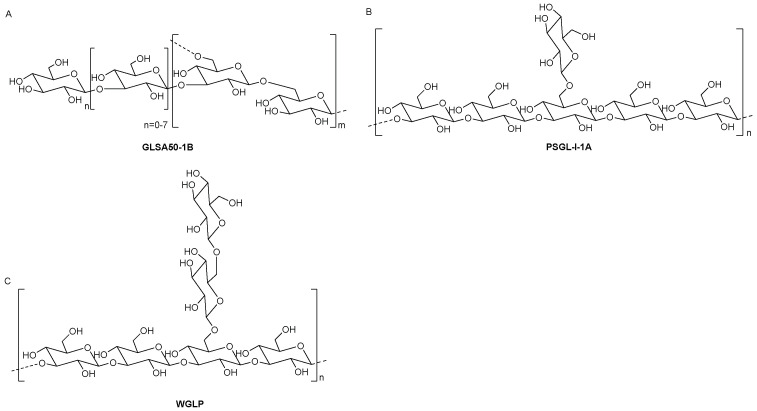
The structures of GLPs. (**A**) GLSA50-1B, (**B**) PSGL-I-1A, (**C**) WGLP.

*Ganoderma lucidum* (*G. lucidum*) is one of the most famous folk medicines in China [162]. Most *G. lucidum* polysaccharides (GLPs) are β-glucans with an MW distribution of 103–106 Da. Ding et al. reported a neutral polysaccharide, GLSA50-1B, with a (1→6) (1→4)-β-D-glucan (Figure 6A) [163]. Fang et al. identified a branched β-D-(1→3)-glucan, named PSGL-I-1A (Figure 6B) [164]. WGLP, a water-soluble polysaccharide, was obtained from spores of *Ganoderma lucidum* (Fr.) Karst and its repeating unit is shown in Figure 6C [165].

Crude polysaccharides from *G. lucidum* work with dacarbazine to inhibit the growth of melanoma tumors [166]. A fucoxylomannan from *G. lucidum* showed effective antiproliferative effects [167]. Ding et al reported that WGLP can significantly inhibit the growth of tumor in vivo at a certain concentration without drug-related toxicity [165]. The water-soluble polysaccharide WSG is effective against lung cancer and tongue cancer [168,169]. In addition, Lin et al. found that the combination of WSG and cisplatin can inhibit cell activity and induce apoptosis [169]. The application of GLPs on gold nanocomposites can be activated effectively for dendritic cells and T lymphocytes in breast cancer-bearing mouse models and inhibit the growth and metastasis of tumors [170]. In addition, GLP-conjugated bismuth sulfide nanoparticles can effectively assist tumor radiotherapy via radiosensitization and dendritic cell activation [171].

## 5. Conclusions

It is predicted that the global number of cancer patients will reach 34 million in 2070, with a doubling of the incidence of all cancers combined relative to 2020 [172]. More and more evidence shows that polysaccharides have great anticancer potential. Polysaccharides are a class of biological macromolecules produced by plants, animals, and fungi, which have received extensive attention in recent years owning to their high therapeutic efficacy and low toxicity. Some polysaccharides isolated from the leaves, seeds, roots, and bark of plants show a certain direct anticancer effect, with mechanisms involved in regulating multiple proteins or signal transduction pathways. Besides, the unique structure diversities and physiochemical properties of polysaccharides lay the foundation for developing various nanocarriers. Drug delivery methods based on polysaccharides nanomaterials help to achieve targeted delivery of immunotherapeutic agents to immune cell subtypes and effectively improve the therapeutic effect of drug carriers. In addition, the degradation products of polysaccharides are normal monosaccharides in vivo and can be recycled by cells without accumulation in the tissue.

In a word, this article reviews the latest progress of polysaccharides and polysaccharide-based nanomaterials and their applications in cancer immunotherapy. The anticancer properties of polysaccharides are mainly mediated through two ways: (I) direct cytotoxicity and (II) as a targeted nano carrier platform, which carries traditional anticancer drugs. Although there are still many unsolved problems in this field, the clinical value and broad application prospects of anticancer polysaccharides make them an important direction of new drug development.

## Figures and Tables

**Table 1:** Performance and structural features of natural anticancer polysaccharides.

Natural Polysaccharides	Performances	Structural Features
Polysaccharides from plants	Target Twist/AKR1C2/NF-1 pathway	acidic protein–polysaccharide
Polysaccharides from animals	Antiangiogenic properties	GlcN-GlcA or GlcN-IdoA
Polysaccharides from fungi	Inhibiting JAK2/STAT3 signaling pathway	β-(1→3) glucose linkages

**Table 2:** Ginseng polysaccharides with antitumour activity.

Compound	Structure Features	MW	Antitumor Mechanism	Ref.
PGPW1	97.4% carbohydrate and 1.2% uronic acid	~3.5 × 105 Da	Not been elucidated	[14,15]
PGP2a	Acidic protein–polysaccharide	~3.2 × 104 Da	Target Twist/AKR1C2/NF-1 pathway	[16]
RG-I	RG-I and side chains AG-I	~6 × 104 Da	Bound to galectin-3	[17]
MCGP-1	The ratio of Rha/GalA is 0.82	1.649 × 105 Da	Might be related to the Ara residues linked to the surface of the polysaccharide	[18]
MCGP-2	Mainly composed of GalA, Ara, Gal, Rha, and Glc	1.644 × 105 Da	The same mechanism as MCGP-1	[18]
MCGP-3	The characteristic compositions of RG-I pectin	1.572 × 105 Da	The same mechanism as MCGP-1 and contains disaccharide [-(1, 4)-α-D-GalAp-(1, 2. -α-L-Rhap-]	[18]
MCGP-4	The characteristic compositions of RG-I pectin	1.673 × 105 Da	The same mechanism as MCGP-1	[18]
MCGP-5	The ratio of Rha/GalA is 0.24	1.600 × 105 Da	The same mechanism as MCGP-1	[18]
MCGP-6	Mainly composed of GalA, Ara, Gal, Rha, and Glc	1.592 × 105 Da	The same mechanism as MCGP-1	[18]
MCGP-7	Mainly composed of GalA, Ara, Gal, Rha, and Glc	1.520 × 105 Da	The same mechanism as MCGP-1	[18]

**Table 3:** Seeds’ polysaccharides with anticancer activity.

Plants Species	Types of Carcinoma Cell Lines	Ref.
Peony seeds	Pc-3/HCT-116/MCF-7/Hela	[45]
*Chenopodium quinoa* seeds	SMMC 7721/MCF-7	[46]
*Psidium guajava* L. seeds	MCF-7	[47]

**Table 4:** Polysaccharides from other species of plants with antitumour activity.

Plants Species	Structure Features	Types of Carcinoma Cell Lines	Ref.
Broccoli	Comprised of Ara, Gal, and Rha with a molar ratio of 5.3:0.8:1.0	HepG2, Siha cervical, MDA-MB-231	[77]
*Gleoestereum incarnatum*	Composed of Gal, Glc, xylose, and Man at molar ratios of 1:4.25:1.14:1.85	HepG2	[78]
*Zizyphus jujuba cv.* *Muzao*	Presence of RG-I domains and typical pectic polysaccharides, with homogalacturonan (methyl and acetyl esterified)	HepG2	[79]
*Taxus chinensis var.* *mairei fruits*		S180	[80]
*Huperzia serrata*	Composed of Gal, Glc, Ara, Rha, Man, GalA, and so on	Skov3 and A2780	[81]
Dandelion	α-type polysaccharides, consisted of Glc, Gal, Ara, arabinose rhamnose, and GlcA	HepG2	[82,83]
*Dendrobium nobile* Lindl	Composed of Gal, Glc, Ara, Rha, Man, and so on	Sarcoma 180	[84]

**Table 5:** Application of HP in antitumour therapy.

Compound	HP Combination Types	Anticancer Mechanisms	Types of Cancer	Ref.
LHT	HP–drug conjugate	Antiangiogenic properties	Pancreatic cancer cells-bearing mice	[99]
Oral LMWH conjugate (LHTD4)	HP–drug conjugate	Antiangiogenic properties	A549 lung cancer cells	[100]
Tinzaparin, a LMWH	HP fragments	Reverses the cisplatin resistance in A2780cis cells	A2780cis cells	[101]
Deoxycholic acid conjugatedHP fragments (HFD)	HP–drug conjugate	Inhibiting VEGF165	SCC7 cells	[102]
LMWH-Suramin	HP–drug conjugate	Inhibiting VEGF165	SCC7-bearing mouse model	[103]
HP-suramin/PEGylated protamine	HP–drug conjugate	Antiangiogenic properties	SCC7-bearing mouse model	[104]
HP-functionalized Pluronic nanoparticles	Polymeric nanoparticles	Antiangiogenic properties and drug combination	Gastric cancers	[105]
Heparin/polyethyleneglycol (PEG) hydrogel	Nanogels	Antiangiogenic properties and drug combination	Breast cancer	[106]
LMWH-poloxamer	Nanogels	Enhancing the efficacies, minimizing the side effects ofdalteparin, and exhibiting a good thermosensitivity	Xenograft S180 sarcoma tumor	[107]
HP-containing cryogel microcarriers	Polyelectrolyte complex nanoparticles	Reversible strong electrostatic interaction	Metastatic breast cancer	[108]
HP-Folate-Tat-Taxol	Polyelectrolyte complex nanoparticles	Negatively charged nanoparticles may cause lower toxic effect	Breastcancer cells	[109]
LMWH–quercetin conjugate	HP–drug conjugate	Antiangiogenic properties	MCF-7 tumor cells	[110]
HP-Poloxamer	HP-coated inorganic nanoparticles	Antiangiogenic properties and drug combination	HeLa cells	[111]
Heparosan-cystamine-vitamin E succinate	Nanogels	Increase tumor selectivity and improve the therapeutic effect	MGC80-3 tumor cells	[112]
LMWH-TOS	Polyelectrolyte complex nanoparticles	Antiangiogenic properties and drug combination	4T1 solid tumor model	[113]
HP–folate–retinoic acid bioconjugates	Polyelectrolyte complex nanoparticles	Drug combination	HeLa cells	[114]
HP-reduced graphene oxide nanocomposites	Polyelectrolyte complex nanoparticles	Combinational chemotherapy and photothermal therapy	MCF-7 and A549cells	[115]
PEGylated HP-based nanomedicines	Polyelectrolyte complex nanoparticles	Photodynamic therapy	4T1 cells	[116]

**Table 6:** Application of HA in antitumour therapy.

Compound	HA Combination Types	Anticancer Mechanisms	Types of Cancer	Ref.
Carbon nanotubes-Chitosan (CHI)-HA-DOX	Polymeric nanoparticles	CD44-targeted, hydrophilic	HeLa cells	[120]
HA-DOX-afatinib-CaP	Polymeric nanoparticles	CD44-targeted, high-densitycarboxyl groups	A549 lung cancer cells	[121]
HA-Curcumin (Cur)	Nanogels	CD44-targeted	A549 lung cancer cells	[122]
HA-Sinulariolide	Polymeric nanoparticles	CD44-targeted	A549 lung cancer cells	[123]
HA-Cur-prodrug-CaP	Polymeric nanoparticles	CD44-targeted	MB-MDA-231 mouse model	[124]
HA-cystamin-pyrenyl-Ir(III)	Polymeric nanoparticles	CD44-targeted, hydrophilic	A549 tumor-bearing mice	[125]
HA-DOX-cisplatin	Nanogels	CD44-targeted	A2780 cell lines	[126]
HA-keratin-DOX	Nanogels	CD44-targeted, negative charge and good hydrophilicity	4T1 and B16 cells	[127]
HA-Pemetrexed	HA–drug conjugate	CD44-targeted, as a prognostic marker in malignant pleural mesothelioma	Malignant pleuralmesothelioma model	[128]
HA-fluvastatin-encapsulating liposomes	Polymeric nanoparticles	CD44-targeted, hydrophilic barrier	Breast cancer stem cellxenografted mouse model	[129]
HA-coated silica/hydroxyapatite- DOX	HA-coated inorganic nanoparticles	CD44-targeted	4T1 tumor-bearing mice	[130]
HA-sclareol/poly-lactic-co-glycolic acid	HA-coated inorganic nanoparticles	CD44-targeted, hydrophilic	MCF-7 and MDA-MB468 cell lines	[131]
HA-coated camptothecin	HA-coated inorganic nanoparticles	CD44-targeted	MDA-MB-231 cells	[132]
HA and poly-(N-ε-carbobenzyloxy-L-lysine)	Polymeric nanoparticles	CD44-targeted	HepG2 tumor-bearing mice	[133]
Ursolic acid-loadedin a poly-L-lysine coat and HA	HA-coated organic nanoparticles	CD44-targeted	SCC-7 xenograft tumor model	[134]
folic acid- and dopamine-decorated HA	HA-coated organic nanoparticles	CD44-targeted	B16 melanoma model	[135]
HA-Cu_2_−_X_S	HA-coated organic nanoparticles	CD44-targeted, biocompatibility	CT26.WT cells-bearing mice	[136]
HA Conjugated ZincProtoporphyrin	HA conjugated cincprotoporphyrin	CD44-targeted	C26 colon cancer cells	[137]
Irinotecan-loaded self-agglomerating HA	Polymeric nanoparticles	CD44-targeted	H23 non-small-cell lung cancer cells	[138]
HA-SuperparamagneticIron Oxide	Polyelectrolyte complex nanoparticles	CD44-targeted	U87MG cells	[139]

**Table 7:** Types of CS.

CS Types	Major Disaccharide Unit	Other Disaccharide Unit
CS-A	GlcA-GalNAc4S	GlcA-GalNAc/GlcA2S-GalNAc
CS-B(DS)	IdoA-GalNAc4S	IdoA2S-GalNAc4S/GlcA3S-GalNAc
CS-C	GlcA-GalNAc6S	IdoA-GalNAc4S6S/GlcA3S-GalNAc4S
CS-D	GlcA2S-GalNAc6S	IdoA2S-GalNAc4S6S/GlcA3S-GalNAc4S6S
CS-E	GlcA-GalNAc4S6S	IdoA2S-GalNAc/GlcA3S-GalNAc6S

## Data Availability

Not applicable.
